# Targets of Neutrophil Influx and Weaponry: Therapeutic Opportunities for Chronic Obstructive Airway Disease

**DOI:** 10.1155/2017/5273201

**Published:** 2017-05-15

**Authors:** Carina Kärrman Mårdh, James Root, Mohib Uddin, Kristina Stenvall, Anna Malmgren, Kostas Karabelas, Matthew Thomas

**Affiliations:** Respiratory, Inflammation and Autoimmunity IMED, AstraZeneca R&D, Gothenburg, Sweden

## Abstract

Neutrophils are important effector cells of antimicrobial immunity in an acute inflammatory response, with a primary role in the clearance of extracellular pathogens. However, in respiratory diseases such as asthma and chronic obstructive pulmonary disease (COPD), there is excessive infiltration and activation of neutrophils, subsequent production of reactive oxygen species, and release of serine proteases, matrix metalloproteinases, and myeloperoxidase—resulting in collateral damage as the cells infiltrate into the tissue. Increased neutrophil survival through dysregulated apoptosis facilitates continued release of neutrophil-derived mediators to perpetuate airway inflammation and tissue injury. Several target mechanisms have been investigated to address pathologic neutrophil biology and thereby provide a novel therapy for respiratory disease. These include neutrophil influx through inhibition of chemokine receptors CXCR2, CXCR1, and PI3K*γ* signaling and neutrophil weaponry by protease inhibitors, targeting matrix metalloproteinases and neutrophil serine proteases. In addition, neutrophil function can be modulated using selective PI3K*δ* inhibitors. This review highlights the latest advances in targeting neutrophils and their function, discusses the opportunities and risks of neutrophil inhibition, and explores how we might better develop future strategies to regulate neutrophil influx and function for respiratory diseases in dire need of novel effective therapies.

## 1. Introduction

Asthma and chronic obstructive pulmonary disease (COPD) are heterogeneous respiratory conditions characterized by airway inflammation, remodeling, and restricted pulmonary air flow—principally distinguished by reversible airway hyperreactivity in asthma. Together, asthma and COPD represent a major proportion of airway disease burden, where asthma affects 235 million people worldwide, COPD affects 384 million people worldwide, and 3 million deaths every year are caused by COPD globally (WHO http://www.who.int/respiratory/copd/en/, [1]). The global prevalence of COPD has been estimated to be 11.7% [2], and the global prevalence of adult asthma has been estimated to be 4.3% [3]. Current therapeutic strategies focus upon symptom relief and control using as-needed short-acting *β*_2_-agonist (SABA), inhaled corticosteroids (ICS), and long-acting *β*_2_-agonist (LABA) for asthma [4] with the addition of long-acting muscarinic antagonists (LAMA) and phosphodiesterase type 4 (PDE4) inhibitors for COPD [5]. Restricted air flow is treated by bronchodilators and the inflammatory response by ICS in well-controlled mild asthma. Despite the use of a broad selection of specific and nonspecific immune regulatory therapies (e.g., ICS, emerging anticytokine antibodies), no treatment other than glucocorticoids targets the underlying cause of inflammation; hence, both asthma and COPD still represent a significant unmet medical need. Indeed, only half of asthma patients respond adequately to current therapies [4].

The most common cause of COPD is cigarette smoking, but some patients develop COPD from inhaling smoke through combustion of biomass fuel or other irritants. Chronic inflammation of the lung, particularly in peripheral airways and parenchyma, is the hallmark of disease in COPD and may be the underlying cause for small airway destruction that progresses with disease. The underlying inflammation then increases during acute exacerbations. COPD is also associated with systemic inflammation which may lead to comorbidities. There is a characteristic inflammation pattern with increased numbers of macrophages, T lymphocytes, and B lymphocytes, together with increased numbers of neutrophils in the airway lumen [6]. The inflammatory response in COPD involves both innate and adaptive immune responses, which are linked through the activation of dendritic cells. While endothelial cells and macrophages are the key cells responsible for triggering the immune response in COPD, classical adaptive immunity is the key driver in asthma. Airway inflammation in asthma is typically associated with Th2 cytokines, produced by activated CD4+ T cells polarized in the presence of interleukin (IL) 4. Cytokines produced by Th2 cells comprise of IL-4, IL-5, and IL-13 [6]. Asthmatic airways exposed to environmental stimuli such as allergens, viruses, pollutants, and bacteria lead to the epithelial damage which activate cells of the innate immune system such as dendritic cells, basophils, mast cells, eosinophils, and macrophages. Dendritic cells then direct the adaptive immune responses, promoting differentiation of Th2 cells and isotype switching of B cells to produce IgE.

However, both severe asthma and COPD, as well as bronchiectasis and cystic fibrosis, also have features of dysregulated neutrophil recruitment, activation, and survival that result in release of toxic proteases and reactive oxygen species perpetuating airway inflammation and tissue injury. Importantly, none of the currently available medical therapies selectively target neutrophils, even though neutrophils appear to have a role in disease pathogenesis and are causative for tissue damage in severe disease [7]. Thus, innovative therapeutic approaches are needed to treat poorly controlled asthma and COPD patients with sustained neutrophilic inflammation.

Neutrophils are the most abundant leukocytes in blood and are part of our native or innate immunity, and together with NK cells, platelets and macrophages, they mainly act as part of our defense to protect against microbes. Specifically, neutrophils are the final effector cells of antimicrobial immunity of an acute inflammatory response, with a primary role in the clearance of extracellular pathogens [8]. Microorganisms and particles reaching the airways and lung evoke a massive influx of neutrophils. However, in airway diseases such as severe asthma and COPD, there is excessive neutrophil recruitment, activation, and defective apoptosis. Neutrophil production of reactive oxygen species and release of serine proteases, matrix metalloproteinases, myeloperoxidase, and lysozymes contribute to lung tissue damage and airway remodeling. COPD and severe asthma are both characterized by sustained neutrophilic inflammation of the airways [7, 9–14], and the number of viable neutrophils in sputum is negatively correlated with lung function as measured by forced expiratory volume in 1 second (FEV1) [13, 15–18].

This review therefore sets out to describe the role of neutrophils in mediating inflammation and tissue damage in obstructive airways diseases and reviews potential therapeutic targets (Table 1) for measuring/modulating neutrophil presence and activity in the lung.

### 1.1. Targeting Neutrophil Influx

#### 1.1.1. Chemokine Receptor Antagonism

There are several proteins involved in the chemoattraction, rolling, tight adhesion, and transmigration of neutrophils. Neutrophil trafficking out of the circulation into the lung is a multistep process, and each step can be targeted by a different mechanism. Neutrophils must first exit the circulation by rolling on the endothelium mediated by selectins, then tight adhesion using integrins, followed by migration via chemokine receptors. Migration into the inflamed tissues of the lung involves both transendothelial and transepithelial migration. During the first step in neutrophil emigration from the circulation, the adhesion to the vascular endothelial cells is mediated by selectins and these are similar between the intestine and lung, for example, L-, E-, and P-selectins, P-selectin glycoprotein ligand, and *α*4*β*1 integrin. Transepithelial migration follows a similar pattern of adhesion, migration, and postmigration events, the difference being that neutrophil adhesion to the epithelium occurs on the basolateral as opposed to the apical surface. In the first stage of transepithelial migration, neutrophils adhere to the basolateral epithelial surface via *β*2 integrins, and in most epithelial cell types, it is mediated via the CD11b/CD18 molecule. CD11b/CD18 is present both in intestinal and in bronchial epithelium while CD11a/CD18 is exclusive to bronchial and alveolar epithelium and CD11c/CD18 exclusive to bronchial epithelium. After firm adhesion to the basolateral surface of the epithelium, neutrophils begin to migrate across the epithelial monolayer through the paracellular space by mechanisms using the cell surface molecules CD47, SIRP*α*, and SIRP*β*. Once the neutrophils have completed migration, they are retained on the luminal side as a defense barrier to clear pathogens [19]. The process is propagated by circulating leukocytes entering into inflamed tissue in response to inflammatory mediators. The process by which neutrophils enter into the tissue are directed through chemotactic processes regulated by several families of proteins including inflammatory cytokines, adhesion molecules, matrix metalloproteases, and chemokines. Four subfamilies of chemokines can act on chemokine receptors that are expressed on different inflammatory cells. For neutrophils, the chemokines GRO*α* (CXCL1) and IL-8 (CXCL8) are potent chemoattractants and activate G protein-coupled receptors (GPCRs) CXCR1 and CXCR2 [20]. In patients with moderate to severe asthma, increased expression of CXCL8 has been shown to correlate with raised neutrophil numbers in sputum, which in turn is associated with an increase in the frequency of exacerbations of acute asthma [21, 22]. Activation of CXCR2 by, for example, CXCL8 mediates migration of neutrophils to sites of inflammation. Neutrophilic airway inflammation has been shown to be significantly reduced in animal studies when antagonizing this receptor. In addition, CXCR1 and CXCR2 are also expressed by other cell types associated with chronic inflammation, including macrophages, lymphocytes, mast cells, dendritic cells, and endothelial cells [23–27]. Ligand binding to CXCR1 is mainly responsible for the degranulation of neutrophils, whereas CXCR2 regulates recruitment of neutrophils from blood into tissues. CXCR2 is a receptor for a number of chemokines such as the GRO family (CXCL1-3) and CXCL8, all of which are elevated in respiratory inflammatory diseases such as COPD, severe asthma, and acute respiratory distress syndrome. CXCR1 and CXCR2 have similar signaling mechanisms [28], and CXCL8 can potentiate several neutrophil functions triggered through both of its receptors, including phosphoinositide hydrolysis, intracellular Ca2+ mobilization, and chemotaxis. However, CXCR1 has been specifically implicated in phospholipase D activation, respiratory burst activity, and the bacterial-killing capacity of neutrophils [29], suggesting that CXCR1 and CXCR2 might have different physiological roles under inflammatory conditions. CXCL8 signals through both CXCR1 and CXCR2 [28]. Furthermore, CXCL1 may play a homeostatic role in regulating neutrophil egress from bone marrow to blood [30]. Therefore, targeting CXCR2 would be expected to effectively reduce neutrophilic inflammation, mucus production, and neutrophil proteinase-mediated tissue destruction in the lung [22].

Several small molecule C-X-C chemokine receptor antagonists have been developed as a potential therapeutic approach for the treatment of inflammatory disease, including repertaxin, navarixin, and danirixin [14] and AZD5069. CXCR2 selective small-molecule antagonists [31] have been shown not to adversely impact neutrophil effector host defense [32, 33]. These are in different stages of drug development and have been shown to reduce neutrophil recruitment to the lung in clinical studies [34–37]. Effects of inhibiting neutrophil recruitment have been shown by clinical biomarkers and endpoints indicative of disease efficacy in cystic fibrosis, severe asthma, and COPD [38–40]. However, O'Byrne et al. showed that 6 months treatment with AZD5069 did not reduce the frequency of severe exacerbations in patients with uncontrolled severe asthma, thereby questioning the role of CXCR2-mediated neutrophil recruitment in the pathobiology of exacerbations in severe refractory asthma [41]. Intriguingly, CXCR2 antagonists seem mainly to be of clinical benefit in patients who have ongoing exposure-induced stimulation of neutrophil recruitment to the lungs, such as oxidative stress due to tobacco smoking [40]. The only active CXCR2 antagonist trial (using danirixin, formerly called GSK-1325756, currently in clinical phase II trials for COPD (NCT02130193, TrialTroveID-208293, and TrialTroveID-267696)) may provide proof of concept efficacy.

#### 1.1.2. PI3K Inhibition

Phosphoinositide 3-kinase (PI3K) family signaling can influence a multitude of cells and pathologic processes, including those in which neutrophils play a dominant role (reviewed Hawkins et al. [42]). Class I PI3K isoforms (*α*, *β*, *γ*, and *δ*) function by phosphorylating PI(4,5)P_2_ to generate PI(3,4,5)P3 at the plasma membrane following receptor engagement [43] and are the most evolved as targets of drug discovery. Whereas PI3K*α* and *β* isoforms are ubiquitously expressed, PI3K*δ* is largely restricted to myeloid and lymphoid cells [44]. PI3K*γ* is expressed highly in myeloid cells downstream of GPCRs and is an important regulator of neutrophil effector responses, thus making both *γ* and *δ* PI3K isoform inhibition the focus of modulating neutrophil movement.

**Figure 1:**
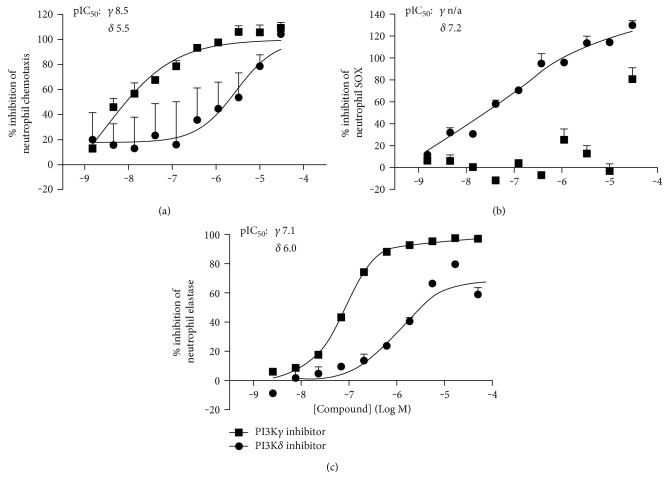
Comparison of PI3K*γ* versus PI3K*δ* inhibition on neutrophil functions. Novel inhibitors with >100-fold selectivity (versus other class 1 PI3K isoforms) for PI3K*γ* (squares) or *δ* (circles) were compared across 3 neutrophil mechanisms. (a) Neutrophil chemotaxis to fMLP. (b) Neutrophil superoxide (SOX) generation following LPS priming and stimulation with fMLP. (c) Neutrophil degranulation (assessed via elastase release) following cytochalasin b priming and stimulation with fMLP. Mean ± standard error of *n* > 3 experiments are plotted as % inhibition. pIC_50_ (−logIC_50_) values for both *γ* and *δ* inhibitors are indicated.

Initial studies used knockout mice to study neutrophils, where Hirsch et al. showed chemoattractant-stimulated PI3K*γ*^−/−^ neutrophils could not produce PI(3,4,5)P3 or downstream activation of pAkt, and displayed impaired respiratory burst and motility [45]. These findings were further confirmed through confocal imaging of knockout neutrophils which indicated PI3K*γ*-mediated control of cell direction via colocalization of AKT and F-actin to the leading edge [46]. A role for PI3K*δ* was discovered in neutrophil migration when trapping of cells in vessels following leukotriene B_4_ (LTB_4_) infusion was observed in PI3K*δ* knockout mice, whereas wild-type controls showed neutrophil transmigration into tissue [47]. The first PI3K*δ*-selective inhibitor studies, using IC87114, also demonstrated blockade of both N-formyl-methionyl-leucyl-phenylalanine- (fMLP-) and tumor necrosis factor-alpha- (TNF-*α*-) induced neutrophil superoxide generation and elastase exocytosis from neutrophils in a mouse model of inflammation [48]. The comparative roles of PI3K*γ* versus *δ* were further investigated in knockout animals of each isoform sensitized with lipopolysaccharide (LPS), indicating a dominant role for PI3K*γ* in neutrophil migration [49]. A key paper from Condliffe et al. made two important observations. Firstly that stimulation of TNF-*α*-primed human neutrophils with fMLP results in biphasic activation of PI3K; the initial phase is largely dependent on PI3K*γ*, whereas the secondary phase is largely dependent on PI3K*δ* (and the first phase itself) [50]. They also showed that murine cells can behave differently to human within their mechanistic systems [50]. Studies from Stephens and colleagues [43] further elucidated roles for PI3K in neutrophil movement, demonstrating PI3K*γ*-mediated PIP_3_ accumulation at the leading edge of the cell to be a vital step in chemokinesis, thus determining the proportion of cells able to move toward a chemokine gradient [51]. Also, studies using both short-term and long-term in vitro neutrophil migration assays showed that PI3K can enhance early responses to the bacterial chemoattractant fMLP, but that it is not required for migration towards this chemoattractant [51]. However, sensing the gradient itself was shown to be PI3K*γ* independent, despite a role for the *γ* isoform in integrin-based adhesion and neutrophil polarization [52]. Yet, a recent bronchiectasis clinical trial where neutrophil chemotaxis was inhibited via CXCR2 antagonism failed to confer therapeutic benefit, thus suggesting that inhibition beyond GPCR/PI3K*γ*-mediated cell movement is needed [37]. It was studies such as these which drove us to investigate our novel PI3K*γ* and PI3K*δ* inhibitors in a human neutrophil chemotaxis assay (Figure 1(a)). Here, we show dose response inhibition curves of low nM potent, >100-fold selective molecules to investigate chemotaxis to fMLP (and other GPCR ligands) and PI3K*γ* versus *δ* isoform signaling. PI3K*γ*-dominated inhibition showed a 3-log advantage in potency, thus confirming the dominance of PI3K*γ* on GPCR-mediated neutrophil movement.

Translational evidence for class 1 PI3K signaling in severe neutrophilic asthma shows that neutrophil chemotaxis triggered by airway epithelial-conditioned media from severe asthmatics can be reduced by a PI3K*γ*-selective inhibitor, whereas the same neutrophil migratory response is insensitive to PI3K*δ* inhibition [53]. However, an inhaled PI3K*δ* inhibitor is currently in early clinical trials for primary immune deficiency, activated PI3K-delta syndrome (APDS) caused by gain of function mutations in PIK3CD, and progressing into both asthma and COPD indications (NCT02294734, ClinicalTrials.gov). The therapeutic hypothesis is based upon rejuvenation of effective directionality in neutrophil movement and therefore a reduction in “collateral damage” observed in a neutrophil with upregulated PI3K*δ* [54]. This hypothesis is intriguing, as it aims to retain effective neutrophil function in the lung and thus minimize any potential for liabilities attributed to immune suppression. The risk of increased infections has been recently identified through a 2016 safety review for idelalisib in three clinical trials, which showed increased numbers of fatal cases related to infections in the treatment arm [55]. Importantly, we are yet to understand the significance of systemic activity of PI3K*δ* inhibitors, thereby affecting lymph node function, versus lung tissue biology and the relative pathologic roles for both PI3K*γ* and *δ* isoforms.

There is clearly an association of chemokine-guided neutrophilic inflammation in disease pathogenesis, but the balance between beneficial control of the disease and maintaining host defense may be limiting the development of drugs targeting chemokine receptors. Alternatively, many complex inflammatory conditions may rely on multiple, interconnected chemotactic stimuli which resist the antagonism of a single pathway. To date, there are only two marketed products targeting chemokine receptors: plerixafor, a small molecule antagonist of CXCR4 used as an immunostimulant in cancer patients, and maraviroc, an antagonist of CCR5 used as treatment of HIV infection [56] despite strong associations of chemokine involvement in disease. Future strategies for inhibiting neutrophil migration may benefit from a more subtle modulatory mechanism aiming to retain host defense (e.g., PI3K*δ* inhibition) or may require a more broad approach targeting multiple stimuli in the lung (e.g., PI3K*γδ* dual inhibition).

### 1.2. Targeting Neutrophil Weaponry

The granules of neutrophils are rich in an array of different antimicrobial molecules that are released in a controlled manner to protect the host from invading pathogens. During chronic neutrophilic inflammation, an increasing number of activated neutrophils secrete granule contents into the extracellular space, where the focal excess of normally protective proteases in the absence of pathogens can become destructive [18]. Intracellularly, neutrophil serine proteases (NSPs) help to destroy ingested bacteria within the phagolysosome. The family of NSPs include neutrophil elastase (NE), proteinase 3 (PR3), and cathepsin G (CG), all located in the primary azurophilic granules, and are together capable of degrading most of the extracellular matrix components such as elastin and collagen [57, 58]. The most studied of these proteases as a drug target is neutrophil elastase, the net activity of which is increased in patients with alpha-1-proteinase deficiency (A1ATD). The genetic loss of this gene results in early-onset emphysema [59]. The hypothesis that COPD is caused by a protease-antiprotease imbalance is further strengthened by studies with exogenous instillation of elastase (or other neutrophil serine proteases) into animal lungs that leads to emphysema [60, 61]. NSPs are amongst the most potent known stimulants of mucus secretion from epithelial cells [62, 63], hypersecretion of which is a common feature across the neutrophilic diseases including cystic fibrosis, bronchiectasis, and chronic bronchitic COPD. Neutrophil elastase may worsen mucus-driven airway obstruction via two processes: activation of the sodium channel ENaC on the apical surface of epithelial cells (via degradation of SPLUNC1, the endogenous inhibitor of ENaC [64]) and indirect degradation of the cystic fibrosis transmembrane conductance regulator (CFTR) [65]. This would lead to dehydration of the airway surface and further weaken the ability of the airways to effectively clear not only mucus but any pathogens present therein.

Of increasing interest is the role of proteinase (PR) 3 in disease, due to the subtle differences in its biological effects. Present in increasing amounts in stable and exacerbating respiratory disease [66], it is capable of influencing the inflammatory milieu by modifying key proinflammatory cytokines such as IL-8, leading to its enhanced stability and potency [67], and release of IL-1*β* and TNF-*α* from monocytic cells [68]. An ever-increasing number of proinflammatory cytokines are being shown to be modulated by not just PR3 [69] but also NE and CG [70]. The inactivation of the IL-6 trans-signaling pathway by NSPs reported by McGreal and colleagues is especially interesting as this mechanism is postulated to be necessary for recruitment of monocytes [71] and neutrophil apoptosis [72], leading to the resolution of inflammation.

Dysregulation of constitutive neutrophil apoptosis may delay the resolution of airway inflammation and is implicated in acute respiratory distress syndrome (ARDS) [73], cystic fibrosis [74], and severe asthma [13] whilst conflicting data exist in COPD [75, 76]. Efferocytosis of apoptotic neutrophils by macrophages is also required for resolution, before they become necrotic and release their cell contents into the inflamed tissue. A significant recognition ligand in this process is the apoptotic neutrophil cell surface-bound phosphatidylserine [77]. Cleavage of this receptor by NE has been reported in vitro using sputum from bronchiectasis and CF patients [78] which may explain why timely clearance of dying neutrophils is defective in the disease. In addition, it has been reported that in vitro NE is capable of creating an “opsonin-receptor mismatch” by cleaving complement receptor 1 (CR1) from the neutrophil surface and C3bi of opsonized *Pseudomonas aeruginosa* [79], impairing clearance of this bacteria commonly found in the CF airway and associated with mortality [80]. An important observation to note is that inhibitors of *Pseudomonas elastase* are reported to not inhibit this degradation in vitro [79]. Additional beneficial effects of blocking NSPs may arise through inhibition of neutrophil extracellular traps (NETs). Formation of NETs has been observed in the airways of patients with asthma [81] and in stable or exacerbated COPD [82, 83]. NET formation itself being an innate immune response can also further affect innate and adaptive immune responses [84, 85]. In addition, NET formation also displays direct cytotoxic effects on alveolar epithelial and endothelial cells [86]. NETs are fibres of chromatin released from neutrophils in an active process named NETosis. Flattening of the cells, chromatin decondensation with histone modifications, and citrullination of histone H3 by peptidylarginine deiminase 4 (PAD4) are a major modification during NETosis and result in DNA released from the cell [87]. Extracellular DNA alters the biophysical properties of mucus and has been correlated with airflow obstruction in CF patients [88].

Links between the neutrophil and the adaptive immune system are being steadily reported, such as inhibition of dendritic cell maturation [89] and the impairment of NK cell activity [90]. Impairment of T cell function via surface antigen cleavage by NSPs [91] could lead to a blunting of the immune response during chronic inflammation. Together, these observations point to the excess neutrophilia and their NSPs potentially having a pivotal role in the cycle of damage and inflammation in neutrophilic respiratory disorders than previously thought.

#### 1.2.1. Neutrophil Elastase Inhibition

A wide variety of synthetic small molecule NE inhibitors have been studied for use in neutrophilic pulmonary disorders with varying degrees of clinical success [92]; however, no compound has progressed further for respiratory indications than phase 2 other than sivelestat which is approved only for acute respiratory indications such as acute respiratory distress syndrome (ARDS). In separate phase 2 trials in bronchiectasis [93], COPD [94, 95], and cystic fibrosis patients [96], the selective NE inhibitor AZD9668 [97] resulted in some beneficial effects, especially in the 4-week bronchiectasis study. Four weeks oral dosing of AZD9668 in these 20 bronchiectasis patients resulted in greatly improved lung function (FEV1 and SVC) and significant decreases in some sputum and plasma inflammatory markers such as IL-6 [93]. These effects were not confirmed in a larger study performed by Bayer (BAY 85-01, NCT01818544, ClinTrials.gov). The effects of another NE inhibitor, MR889, in a small COPD study resulted in no overall changes in the levels of lung destruction markers, but a subset of treated subjects (having shorter than average disease duration of 13.7 years) showed lower urinary desmosine, a marker of elastin degradation [98]. Due to adverse liver effects, another NE inhibitor ONO-6818 was stopped in phase 2. The limited clinical success of NE inhibitors may be in part due not only to inadequate patient phenotype selection but also to the inability to attain stoichiometric equivalent ~mM concentrations of inhibitor at the sites of neutrophil degranulation within the tissue. This issue, coupled with the presence of exclusion zones created when neutrophils are in close contact with extracellular matrix [99], may be solved by inhibiting the protease activation before neutrophils are released into the circulation, rather than inhibit the protease activity. Neutrophil serine proteases are activated early in the promyelocyte stage of neutrophil development via cleavage of a dipeptide, by the cysteine protease dipeptidyl peptidase 1 (DPP1, also known as cathepsin C [100]). Redundancy is absent in this process as illustrated by individuals with inactivation mutations in the gene encoding DPP1, leading to the absence of NSPs [101]. Interestingly, neutrophils from these Papillon-Lefèvre syndrome (PLS) patients who show no generalised immonodeficiency seem incapable of forming NETs [102].

Only two potent and selective DPP1 inhibitors, AZD7986 (NCT02303574, ClinTrials.gov) and GSK2793660 (NCT02058407, ClinTrials.gov), have entered clinical development. Preclinical studies with AZD7986 showed decreased NSP activities in differentiating primary human neutrophils in vitro and in bone marrow neutrophils from treated rats in vivo [103]. In a recent study, DPP1 was found in bronchoalveolar lavage fluid (BALF) from CF patients and patients with neutrophilic asthma as well as in LPS treated macaques but was absent in healthy individuals and untreated macaques [98], the functional significance of which is as yet unknown.

#### 1.2.2. Matrix Metalloprotease (MMP) Inhibition

MMPs, including the highly neutrophil-expressed MMP-8 (neutrophil collagenase) and MMP-9 (gelatinase B), have also been proposed to be involved in the pathophysiology of COPD [104–107]. In the healthy lung, MMPs regulate extracellular matrix turnover and can degrade matrix components such as elastin [108], but again, an excess of these proteases or the cells producing them leads to tissue destruction. It may be that MMPs from other sources may play a more significant role in the development of respiratory diseases such as MMP-12 from macrophages [109] or MMP-7 from hyperplastic epithelial cells in idiopathic pulmonary fibrosis [110, 111]. Whilst many MMPs are expressed by other immune and structural cells, often in greater amounts, the excessive active neutrophilia present in certain chronic lung disorders would add to an increasingly destructive and inflammatory proteolytic milieu. The protease-antiprotease balance might also be adversely altered by the degradation of endogenous MMP inhibitors, such as tissue inhibitor of MMPs (TIMPs), by NE [112]. There are also further possible interconnections between NSPs and MMPs, such as the inactivation of alpha-1-proteinase by MMP-9 [113] and the activation of MMP-9 by NE [114]. Less is known of the role of MMPs in other respiratory disease such as asthma, with MMP-9 and MMP-12 being reported to increase in the airway smooth muscle of fatal asthmatics [115] and mouse knockout studies indicating that several MMPs may be involved in fibrosis [116, 117]. Efforts to develop MMP inhibitors as therapeutic agents have been largely focused outside of respiratory disease and have proved fruitless, largely due to lack of efficacy or the musculoskeletal toxicity that has limited the clinical utility of unselective MMP inhibitors. In a short exploratory study, the dual MMP-9 and MMP-12 inhibitor AZD1236 provided no clinical benefit in moderate/severe COPD patients [118]. However, due to the mechanism of action, significant changes in lung function would not be expected over this time scale in such a small number of stable COPD patients.

#### 1.2.3. PI3K Inhibition

The roles of PI3K*γ* and *δ* isoforms have also been investigated neutrophil degranulation. In Figures 1(b) and 1(c), we show dose-response inhibition curves of low nM potent, >100-fold PI3K-selective molecules to investigate superoxide generation and elastase release, respectively. Interestingly, we saw superoxide generation following LPS priming and stimulation with fMLP was heavily dependent upon PI3K*δ* activity. However, neutrophil degranulation assessed via elastase release following cytochalasin b priming and stimulation with fMLP proved to be a PI3K*γ*-dominated process. And thus, it seems that the differential use of PI3K*γ* and *δ* isoforms is dependent on the priming and the stimuli used. These data build upon a wealth of literature which point toward the value of dual PI3K*γδ* inhibition for the treatment of neutrophil-mediated pathology.

Disease applications for PI3K*γ* &/or *δ* inhibitors span those for which neutrophils are important and beyond—a reflection of the pleiotropic effects anticipated for such molecules. So far, oral systemic inhibitors of PI3K*δ*, exemplified by idelalisib developed for oncology, show target-related toxicity primarily in the gut which hinders therapeutic utility [119]. One could further postulate therapeutic benefit in other pulmonary diseases from neutrophil-mediated bronchiectasis, where sputum neutrophil elastase activity is a biomarker of disease severity [120]. Furthermore, autoimmune activation of neutrophils in Churg-Strauss syndrome has been shown to be PI3K*γ* dependent [121]. However, given our evolving mechanistic understanding of PI3K isoforms in neutrophil function, such diseases would gain far greater therapeutic benefit from inhibition of both PI3K*γ* and *δ* together, where PI3K*δ* controls release of neutrophil stimuli and PI3K*γ* reduces responsiveness to them. Indeed, initial attempts to generate PI3K*γδ* dual inhibitors for inhalation have shown some preclinical success. Doukas et al. induced lung neutrophilia via chronic smoke administration in mice—steroid resistant pathology which could be attenuated by aerosolized TG100-115 [122]. The forthcoming generation of PI3K inhibitors look to improve both potency and selectivity in order to offer a novel therapeutic option for neutrophil-driven diseases. An inhaled PI3K*δ* inhibitor is currently in early clinical trials for activated PI3K delta syndrome (APDS) caused by gain of function mutations in PIK3CD, with the intent of expanding into both asthma and COPD indications.

## 2. Conclusions and Future Outlook

The current therapeutic pharmacological target paradigm for asthma and COPD is not adequately controlling disease in many patients. There is a need for innovative therapeutic approaches to treat severe disease and ultimately modify the underlying pathological changes in asthma and COPD. Although neutrophils appear to play a pathogenic role in severe disease, no neutrophil targeting approaches have been approved to date. Modulating the activity and numbers of neutrophils locally in the affected organs and systemically has been suggested for several chronic inflammatory conditions (e.g., asthma, ulcerative colitis, and rheumatoid arthritis).

Emerging evidence points to the existence of distinct neutrophil subsets in humans that could be phenotypically discriminated based on the surface expression of the markers, Fc*γ*RIII (CD16) and L-selectin (CD62L). Mature neutrophils (CD16^bright^/CD62L^bright^) display a normal-shaped nucleus, immature neutrophils (CD16^dim^/CD62L^bright^) have a banded-shaped nucleus, whereas neutrophils with a hypersegmented shape have a diminished expression of CD62L (CD16^bright^/CD62L^dim^) [123]. Whilst the mature phenotype was found to display a proinflammatory potential, the hypersegmented neutrophils were shown to suppress T cell proliferation in a Mac-1 and H_2_O_2_-mediated fashion and, therefore, may possess a potential immunomodulatory role [123]. It has been speculated that selective blockade of a specific neutrophil subset, notably the disease-promoting mature phenotype, without impacting on the immunoprotective hypersegmented phenotypes, could preserve neutrophil-mediated host-protective immunity [124].

Clinical challenges in using a neutrophil-targeted therapeutic approach have been related to concerns of compromising the patients host defense with an associated increased risk of serious sequelae on opportunistic infections. Furthermore, the unresolved question of whether neutrophils are principal pathogenic drivers or bystanders in more complex inflammatory conditions has also resulted in less effort to target neutrophils selectively. Clearly, reduced neutrophil migration has been shown to reduce hazard exacerbation risk in COPD patients [40]. Significant effect was shown on time to first exacerbation and lung function (FEV1) after 6 months treatment using a 50 mg dose of navarixin, but only in a subpopulation of current smokers, and no effect was shown in the broad COPD population. A possible explanation for response only in active smokers is not clear, and it is conceivable that neutrophils are actually doing their intended job in such circumstances. Furthermore, clear dose-response relationships have been difficult to show and significant dropout of patients at higher doses due to reduction of neutrophil count in blood impacts data interpretation. Local inhibition of neutrophil function (PI3K*γ*/*δ* antagonism) or strategies which spare host defense mechanisms (PI3K *δ* antagonism) may offer effective neutrophil-targeted therapies in the future.

**Figure 2:**
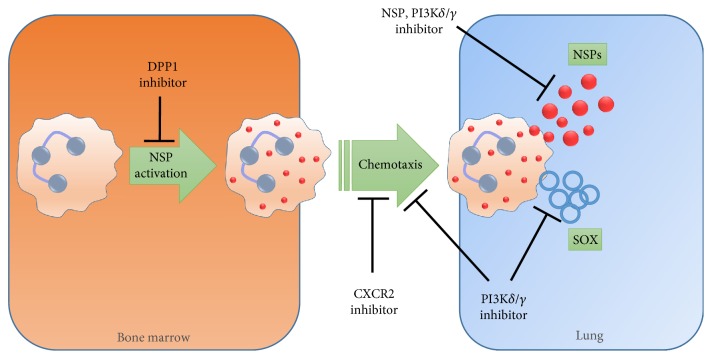
Summary illustration of neutrophil targets in chronic lung disease. Activation of NSPs during neutrophil maturation in the bone marrow is via DPP1. Chemotaxis to the lung can be modulated by targeting CXCR2 or PI3K*δ*/*γ*, the latter of which can also inhibit SOX and NSP release.

Another explanation may be that antineutrophil therapies (illustrated in Figure 2()) need an environment of active damage/challenge to show efficacy. Chronic bronchitic COPD patients have been linked to active smoking and neutrophilic airway inflammation. Chronic cough and sputum production are present in the majority of COPD patients (74.1% of COPD patients) [125] and are associated with frequent exacerbations and hospitalizations. Therefore, selecting patients such as these may improve success in therapeutic development.

In conclusion, targeting the neutrophil weaponry by blocking the activation of proteases via DPP1 inhibition, or neutrophil-mediated NETosis, or multiple neutrophil functions via dual blockade of PI3K*γδ* may show promise as future therapies to address such pressing unmet medical needs.

## Figures and Tables

**Table 1:** Overview of key neutrophil related targets with association to chronic respiratory disease as potential therapeutic targets.

Target	Drug name	Selectivity	Company	Indication	Last reported status	Reference	Subjects	Duration (weeks)
CXCR2	AZD5069	CXCR2	Astrazeneca	Asthma	Phase 2	NCT01704495	640	26
				Bronchiectasis	Phase 2	NCT01255592	52	4
	Danirixin	CXCR2	Glaxosmithkline	COPD	Phase 2	NCT02130193	102	2
	Elubrixin	CXCR2	Glaxosmithkline	CF	Phase 2	NCT00903201	146	4
	Navarixin	CXCR1/2	Merck	Asthma	Phase 2	NCT00632502	37	4
				Asthma	Phase 2	NCT00688467	19	1.3
				COPD	Phase 2	NCT01006616	616	102
	QBM076	CXCR2	Novartis	COPD	Phase 2	NCT01972776	48	8
	SX-682	CXCR1/2	Syntrix	Asthma	Preclinical			

DPP1	AZD7986		Astrazeneca	COPD	Phase 1	NCT02303574	237	4
	GSK2793660		Glaxosmithkline	Bronchiectasis	Phase 1	NCT02058407	33	2

MMP	AZD1236	9/12	Astrazeneca	COPD	Phase 2	NCT00758706	55	6
	AZD2551	12	Astrazeneca	COPD	Phase 1	NCT00860353	81	2
	AZD3342	8/9/12	Astrazeneca	COPD	Phase 1		49	2
	RBx 10017609	12	Glaxosmithkline & Ranbaxy	COPD	Phase 1			

NE	AZD9668		Astrazeneca	Bronchiectasis	Phase 2	NCT00769119	38	4
				CF	Phase 2	NCT00757848	56	4
				COPD	Phase 2	NCT00949975	838	12
				COPD	Phase 2	NCT01023516	615	12
	BAY 85-8501		Bayer	Bronchiectasis	Phase 2	NCT01818544	94	4
	ONO-6818		Ono	COPD	Phase 2			

PI3K	GSK2269557	*δ*	Glaxosmithkline	Asthma	Phase 2	NCT02567708	50	4
				COPD	Phase 2	NCT02294734	126	4
				COPD	Phase 2	NCT02522299	35	12
	GSK2292767	*δ*	Glaxosmithkline	Asthma	Phase1	NCT03045887	44	2
	IPI-145	*δ* (/*γ*)	Infinity	Asthma	Phase 2	NCT01653756	46	2
	RV1729	*δ* (/*γ*)	RespiVert	Asthma	Phase 1	NCT01813084	63	2
				Asthma	Phase 1	NCT02140320	49	4
				COPD	Phase 1	NCT02140346	48	4
	RV6153	*δ* (/*γ*)	RespiVert	Asthma	Phase 1	NCT02517359	55	4
